# Effects of Dill (*Anethum graveolens*) Essential Oil and Lipid Extracts as Novel Antioxidants and Antimicrobial Agents on the Quality of Beef Burger

**DOI:** 10.3390/foods13060896

**Published:** 2024-03-15

**Authors:** Milo Mujović, Branislav Šojić, Tatjana Peulić, Sunčica Kocić-Tanackov, Predrag Ikonić, Danica Božović, Nemanja Teslić, Miloš Županjac, Saša Novaković, Marija Jokanović, Snežana Škaljac, Branimir Pavlić

**Affiliations:** 1 Faculty of Technology Novi Sad, University of Novi Sad, Bul. Cara Lazara 1, 21000 Novi Sad, Serbia; mujovic.9.21.d@uns.ac.rs (M.M.); sojic@tf.uns.ac.rs (B.Š.); suncicat@uns.ac.rs (S.K.-T.); danica_bozovic@live.com (D.B.); marijaj@tf.uns.ac.rs (M.J.); snezana.savatic@tf.uns.ac.rs (S.Š.); 2 Institute of Food Technology, University of Novi Sad, Bul. Cara Lazara 1, 21000 Novi Sad, Serbia; tatjana.peulic@fins.uns.ac.rs (T.P.); predrag.ikonic@fins.uns.ac.rs (P.I.); nemanja.teslic@fins.uns.ac.rs (N.T.); milos.zupanjac@fins.uns.ac.rs (M.Ž.); 3 Department of Food Quality and Sensory Science, Teagasc Food Research Centre Ashtown, D15 DY05 Dublin, Ireland; sasa.novakovic@teagasc.ie

**Keywords:** dill essential oil, dill extracts, burger, quality, shelf life

## Abstract

Dill (*Anethum graveolens* L.) essential oil (DEO) obtained by hydrodistillation (HD) and lipid extracts (DSE_1_ and DSE_2_) obtained by supercritical CO_2_ extraction (SFE) were used as potential antioxidants and antimicrobial agents in beef burgers at two different concentrations (0.075 and 0.15 µL/g). The chemical profile of the lipid extracts and their in vitro antimicrobial activity against the common pathogens *E. coli* and *L. monocytogenes* (MIC and MBC) were determined. The quality and shelf life of the burgers were monitored through (lipid oxidation—TBARS test; protein oxidation—thiol group content and selected biogenic amine content) and microbiological quality (*Enterobacteriaceae*—EB, aerobic mesophilic bacteria—TAMB, lactic acid bacteria—LAB). Dill lipid extracts (DEO and DSE_1_) significantly (*p* < 0.05) reduced lipid oxidation and protein oxidation in beef burgers, while the lipid extract (DSE_2_) showed pro-oxidative effects. The strongest antimicrobial potential against EB was found in SFE_1_150 (1.15 log cfu/g). Putrescine, cadaverine, histamine, and tyramine were not detected in any of the analyzed samples during the storage period, while the total content of biogenic amines ranged from 21.4 mg/kg to 285 mg/kg. Generally, it can be concluded that dill essential oil (DEO) and extract DSE_1_ can be used as novel natural additives in minced-meat products.

## 1. Introduction

The high protein content present in meat, with essential biological potential and significant amounts of minerals and vitamins, is appropriate for meeting the nutritional needs of humans. As a result, as the human population increases, the need for meat and meat products also increases [1]. Depending on the ingredients used, various products can be produced from minced meat: burgers, fresh sausages, and various shaped or unshaped meat products. The ingredients can be conventional (e.g., salts, additives, spices) or novel (e.g., essential oils, fibers) [2,3]. Considering its low price and high nutritional value, this group of meat products is dominant in the fast-food market, making it the most accessible food worldwide [4]. On the other hand, as meat and meat products represent complex systems with a rich nutritional composition, including high-quality proteins, fat-soluble vitamins, minerals, and bioactive compounds, they are very susceptible to various types of spoilage, primarily chemical and biological spoilage [1,5].

Lipid oxidation is one of the leading causes of meat spoilage. It has an unfavorable effect on the color, texture, nutritional value, odor, and taste of the product, which are the main reasons for consumers’ negative sensory quality assessment [5,6]. While extensive research has focused on lipid oxidation in recent decades, protein oxidation remains relatively understudied [7]. Nonetheless, its influence on the quality of meat and meat products has been recognized. This influence is observed from both physicochemical and technological perspectives (e.g., texture, color, taste, water-binding capacity, protein solubility, protein gel formation ability, and protein emulsifying ability), as well as from a nutritional standpoint (e.g., loss of essential amino acids, production of toxic compounds, and reduced bioavailability and digestibility [7]. According to the current Serbian regulations, the use of synthetic antioxidants (e.g., butylated hydroxyanisole—BHA, butylated hydroxytoluene—BHT, t-butylhydroquinone—TBHQ), preservatives (e.g., nitrites, nitrates), and phosphates [8] are not allowed in the production of minced meat products. Therefore, due to intensive manipulation of chilled raw materials, extensive mincing, the absence of additives such as preservatives and synthetic antioxidants, and the unavailability of optimal preservation methods such as various thermal processing techniques, drying, and fermentation, minced meat products exhibit a limited shelf-life [2,9]. In this regard, the relatively high water activity and elevated total bacterial count in chilled meat accelerate the development of chemical and microbiological spoilage, leading to the formation of unacceptable sensory characteristics and potentially increasing the risk of foodborne diseases [10]. From a hygiene–toxicological perspective of meat quality, the identification and quantification of present microbial strains is crucial. The most common causes of spoilage in unpackaged red meat are aerobic bacteria from the family *Pseudomonadaceae* and the genera *Pseudomonas* and *Acinetobacter* [11]. Considering this, during the spoilage of meat and meat products, biogenic amines accumulate as products of bacterial enzymatic decarboxylation of free amino acids. The quantity and type of biogenic amines formed depends on the nature of the substrate, processing conditions (temperature, availability of oxygen, redox potential, pH value, presence of carbohydrates), and types of microorganisms [12,13].

Regardless of the fact that the use of synthetic antioxidants is not allowed, their application is generally associated with toxic and carcinogenic effects. Both consumers and the professional community acknowledged the pressing need for the exploration and application of “bio-preservatives” [14,15]. In this context, natural antioxidants and antimicrobial agents from the group of EOs and various types of plant extracts (e.g., lipid extracts), which are generally recognized as safe (GRAS), represent a reliable alternative to conventional ingredients in the production of minced-meat products [16,17]. The functionality of the usage of plant extracts is based on the bioactive potential of present components (e.g., terpenes, phenols, carotenoids, tocopherols), which are extracted from various aromatic and medicinal plants using different extraction techniques [15]. Dill (*Anethum graveolens* L.) is an annual aromatic plant that belongs to the family *Apiaceae* (*Umbelliferae*). While native to the Eastern Mediterranean and Western Asia, it is now cultivated worldwide and has become naturalized in some parts of Europe and North and South America [18,19,20]. Dill essential oil (DEO) possesses exceptional antimicrobial and antioxidant potential. In the study of Behbahani et al. [21], the influence of edible coatings (PSMS) for beef meat (active packaging films) treated with different concentrations of DEO was investigated. DEO is produced by hydrodistillation (HD), which is, on the one hand, economically viable and the most commonly used extraction method. On the other hand, it has certain drawbacks (e.g., deviation in the chemical composition of the obtained EOs, non-standard quality and yield, high energy consumption during the heating and cooling of the obtained vapors, irrational duration of the process, and poor selectivity towards targeted bioactive compounds EOs) [15,22]. In order to overcome the mentioned drawbacks of conventional extraction techniques, one of the novel techniques, supercritical fluid extraction (SFE), is seeing increasing application [15,23]. In addition to achieving higher yield, standard quality, and exceptional selectivity towards desired bioactive compounds, the use of organic solvents is notably absent with the use of SFE (e.g., CO_2_), which is considered inert and non-toxic (generally recognized as safe—GRAS), falling into the category of “green” techniques for obtaining EOs and lipid extracts) [15,24,25]. Beyond the discussed quality parameters of the extracted EOs, it is essential to consider the economic aspect of application in the meat industry. Despite the significantly higher initial economic investments with SFE, the cost of the obtained lipid extracts is economically competitive compared to EOs and, in some cases, even cheaper than the application of conventional extraction technique (HD) [2].

It should be highlighted that one of the main challenges in modern minced-meat processing is the development and application of emerging natural extracts with potent antioxidant and antimicrobial activity and GRAS status. In this regard, EOs and lipid extracts obtained by the novel SFE extraction technique could be a good solution. Since the current scientific knowledge regarding the application of dill and its extracts as novel additives in meat processing is very scarce, additional research is needed. Thus, this study aims to examine the antioxidant and antimicrobial potential of DEO isolated by conventional hydrodistillation and dill lipid extracts obtained by SFE in fresh beef burger processing.

## 2. Materials and Methods

### 2.1. Chemical and Reagents

Commercial carbon dioxide (Messer, Novi Sad, Serbia) with >99.98% (m/m) purity was used for laboratory-scale supercritical fluid extraction. 2-Thiobarbituric acid, Trichloroacetic acid; Butylhydroxytoluene (BHT), n-Hexane, 1,1,3,3-Tetraethoxypropane, Ellmans reagent (DTNB), and Bovine serum albumin (BSA), all of analytical grade, were purchased from Sigma-Aldrich GmbH (Darmstadt, Germany). l-cysteine of analytical grade was purchased from Fisher Scientific (Loughborough, UK). The standard compounds for HPLC analyses, Tryptamine hydrochloride, 2-Phenyethyamine hydrochloride, Pytrescine dihydrochloride, Cadaverine dihidrochloride, Histamine dihydrochloride, Tyramine hydrochloride, and 1,7—diaminoheptane, were purchased from Sigma-Aldrich GmbH (Darmstadt, Germany). Thymol, *trans*-anethol, (+)-borneol, (−)-borneol, α-terpineol, L-carvone, (R)-(+)-limonene, eucalyptol, farnesol, neryl acetate, (±)-citronellal, citral, γ-terpinene, nerol, α-pinene, p-cymene, (−)-*trans*-caryophyllene, geraniol, geranyl acetate, carvacrol, eugenol, sabinene hydrate, bornyl acetate, linalyl acetate, myrcene, (±)-camphor were purchased from Sigma-Aldrich (St. Louis, MO, USA). All other chemicals used were of analytical reagent grade.

### 2.2. Plant Material and Extract Preparations

Dill (*Anethum graveolens*) was obtained from an agricultural holding, Bačko Novo Selo (Bačko Novo Selo, Serbia). Harvesting was performed by hand at the stage of full maturity in the summer of 2019. After harvesting, the plant material was stored in paper bags at room temperature until needed for further analysis. Detailed processing and handling conditions of the plant material are described in our previous work [26].

The dried plant material was ground in a household blender and the mean particle size of the sample (0.3358 mm) was determined by sieve sets (CISA, Cedaceria Industrial, Barcelona, Spain). The moisture content of the plant material was analyzed by drying the plant sample at 110 °C until constant weight and 8.49% moisture content was observed in the sample.

Dill essential oil (DEO) was isolated by the official *Ph. Eur. VII* procedure [27], which was described in detail in our previous work [2]. On the other hand, supercritical fluid extraction was used as a green and environmentally friendly method for isolation of lipid extracts. DSE_1_ and DSE_2_ were obtained in the following set of conditions: 100 bar and 40 °C and 300 bar and 40 °C, respectively, while all other parameters were held constant. SFE method, as well as the properties of the SFE processing plant, were described in detail in a previous work [2]. The observed yields of DEO, DSE_1_ and DSE_2_ were 4.59%, 4.89%, and 7.03%, respectively. All extracts and essential oils were analyzed by gas chromatography–mass spectroscopy using a previously described methodology [28] and the results were expressed as relative percentages (%) ± standard deviation (Std).

### 2.3. Antimicrobial Activity of DEO and Dill Lipid Extracts

The minimal inhibitory concentration (MIC) and minimal bactericidal concentration (MBC) of DEO and dill lipid extracts obtained by SFEs were determined using the broth microdilution method described in our previous study [29]. The antimicrobial activity of the aforementioned extracts was evaluated against *Escherichia coli* ATCC 8739 and *Listeria monocytogen* ATCC 13932 obtained from the American Type Culture Collection. All tests were conducted in duplicate for each natural extract.

### 2.4. Beef Burger Processing

Beef burger processing was described in detail in our previous study [2]. Dill essential oil (DEO) and lipid extracts (DSE_1_ and DSE_2_) were added at two different concentrations (0.075 and 0.150 µL/g): DEO (DEO75, DEO150), DSE_1_ (DSE_1_75, DSE_1_150) and DSE_2_ (DSE_2_75, DSE_2_150) were incorporated into the basic formulation of beef burgers. Each beef burger, weighing approximately 0.1 kg, was placed in a cooling chamber at 3 ± 1 °C for a duration of three days. Samples were collected at various intervals (0, 1, 2, and 3 days) from both the treatment and control groups (without DEO and dill lipid extracts), with each sample consisting of three randomly selected beef burgers. Physicochemical and microbiological analyses were performed on three samples from each group, with duplicate measurements.

### 2.5. Physicochemical Characteristics of Beef Burgers

The pH was measured directly using a digital pH meter (Testo 205, West Chester, Pennsylvania, USA) calibrated with standard buffers (pH = 4.00 ± 0.05; pH = 7.00 ± 0.01 at 20 ± 2 °C). The surface of fresh beef burgers was analyzed for color using the MINOLTA Chroma Meter (Model CR-400) with an 8 mm aperture in the measuring head and standard additions to measure CR-A33b (Konica Minolta Inc., Osaka, Japan). The lighting conditions were D-65, and a standard observer angle of 2° was utilized. Prior to each set of measurements, the instrument was calibrated using a white ceramic tile. The color characteristics were expressed using the CIE Lab* system, with L* representing lightness, a* representing redness and greenness, and b* representing yellowness and blueness [30]. Lipid oxidative reactions in beef burgers were assessed using the TBARS test as described in [31], with results expressed as mg malondialdehyde (MDA) per kg of beef burger. Protein oxidation was determined by measuring free thiol group contents (nmol of thiol per mg of protein) following the method described by [32].

### 2.6. Microbiological Quality of Beef Burger

The TAMB (total aerobic mesophilic bacteria count), LAB (lactic acid bacteria count), and EB (total Enterobacteriaceae count) were determined by the standard ISO procedures, which were described in detail in our previous study [2]. The results were expressed as a log CFU/g.

### 2.7. Biogenic Amines Determination

Six biogenic amines (tryptamine, phenylethylamine, putrescine, cadaverine, histamine, and tyramine) were determined as their dansyl derivatives following the high-performance liquid chromatography. Sample preparation and extraction were performed according to Eerola et al. [32]. HPLC analysis was performed by using liquid chromatography (Agilent 1200 series) equipped with a diode array detector (DAD), Chemstation Software B.03.02. (Agilent Technologies, Santa Clara, CA, USA), a binary pump, an online vacuum degasser, an auto sampler, and a thermostated column compartment on an Agilent, Eclipse XDB-C18, 1.8 μm, 4.6 × 50 mm column. Solvent gradient was performed by varying the proportion of solvent A (acetonitrile) and solvent B (water). The flow rate was 1.5 mL/min., the column temperature was 40 °C, and 5 μL of sample was injected. All analyses were performed on three sample sausages from each batch, in duplicate. The detection limits of the amines were determined to be 0.10 mg/g for putrescine, 0.17 mg/g for cadaverine and tyramin, and 0.25 mg/g for tryptamine, phenylethylamine, and histamine.

### 2.8. Statistical Analysis

Statistical analysis was conducted using STATISTICA 14.0 (TIBCO Software Inc., Palo Alto, CA, USA). All data were presented as mean values with their standard deviation indicated (mean ± SD). Variance analysis (ANOVA) was performed, with a confidence interval of 95% (*p* < 0.05). Means were compared by a post hoc Duncan test.

## 3. Results and Discussion

### 3.1. Chemical Composition of Applied Essential Oils and Lipid Extracts

GC/MS analysis identified and quantified the main compounds of EO and dill lipid extracts obtained by hydrodistillation and supercritical fluid extraction. A total of 17 different compounds were identified, representing more than 99.98% of the total amount of EO and 99.99% of the total dill extracts. The obtained EO and lipid extracts revealed that the main groups of compounds were monoterpene hydrocarbons and oxygenated monoterpenes. The chemical compositions of the DEO and DSE extracts of *Anethum graveolens* are represented in Table 1.

The most dominant compounds in DEO obtained by HD were carvone (47.91%) and limonene (46.26%), while in DSE, the same compounds stood out with a slight difference in relative percentages compared to EO. In the DSE_1_ extract obtained under conditions of pressure 100 bar, temperature 40 °C and CO_2_ flow rate 0.3 kg/h, the percentage of limonene was 44.03%, while the percentage of carvone was marginally higher in comparison with the second extract (50.04%). The DSE_2_ extract (300 bar, 40 °C, 0.3 kg/h) showed that the relative percentage of limonene was 46.64% and that of carvone was 47.62%. By comparing the supercritical extraction at different conditions, it can be concluded that the chemical profile among the samples does not differ, except that limonene is isolated in a slightly higher percentage in DSE_2_ compared to DSE_1_. Other compounds that have been isolated by HD and SFE include *trans*-dihydro carvone (*trans*-p-Menth-8-en-2-one), *cis*-dihydro carvone (*cis*-p-Menth-8-en-2-one), α-phellandrene, myrcene and p-cymene in different relative percentages. The percentages of these isolated compounds show negligible differences both between techniques and between different SFE conditions. *cis*-Limonene oxide is a compound that is isolated only by HD. According to the literature, the most abundant components obtained through HD from dill were limonene and carvone in varying proportions. This aligns with the findings reported in the literature data [33,34,35,36,37]. Garcez et al. [38] performed both SFE and HD to obtain the chemical profile of *Anethum graveolens*. In the case of HD, the main compounds isolated in EOs were carvone (34.801%) and dill apiole (31.029%), while other compounds, such as limonene and *trans*-dihydro carvone, were present in lower percentages. These results are partially in accordance with the results of this paper. SFE extraction was performed at a pressure of 100 bar, a temperature of 55 °C, and a flow rate of 1000 g/h. The main compounds isolated under these conditions were dill apiol (77.931%) and carvone (12.654%). The main difference in the results is the compound dill apiol, which was one of the dominant compounds compared in this work, in which dill apiol was not isolated at all. Additionally, limonene was isolated in a very small amount compared with the results of this work. Similar results were obtained by Garcez [38] using SFE under different conditions, with the best results achieved at a pressure of 100 bar and a particle diameter of 0.5 mm. The obtained extract consisted of 84.58% of dill apiol and 10.95% of carvone. In terms of HD, at the smallest diameter of 0.5 mm, the highest proportion of dill apiol (63.11%) was obtained, while the percentage of carvone was 22.89%. According to Li et al. [39], the EO of dill seeds was obtained by SFE under the following conditions: 20 MPa, 40 °C, and CO_2_ flow rate 25 L/h. The most abundant compound was D-carvone, with a content of 40.36%, consistent with the findings of this study. Other isolated compounds in slightly smaller percentages were D-limonene (19.31%) and apiol (17.50%). In another study, a group of authors investigated the chemical profile of dill seeds (*Anethum sowa*) from India using CO_2_ extraction and hydrodistillation [40]. They applied different CO_2_ extraction conditions, where the best parameters were 35 °C and 25 MPa, and a density of 0.88 g/cm^3^. The predominant compounds in SFE extracts were dill apiole, dihydrocarvone, and limonene. In the case of HD, the chemical composition was the same, but the principal compound was limonene, followed by dihydrocarvone. The difference between these results and the results in this paper may be due to differences in geographical origin and growing conditions. Babri et al. [41] performed hydrodistillation on dill seed in order to identify and quantify the obtained EO. The components that were present in the highest percentages were R-(-)-carvone, apiol, and limonene (38.899%, 30.812%, and 15.938%, respectively) [28]. The largest amount of essential oil was isolated from the fruit, where the dominant compounds were carvone (75.21%) and limonene (21.56%), which is in accordance with the results of this research.

### 3.2. Minimal Inhibitory Concentration (MIC) and Minimal Bactericidal Concentration (MBC) of the Applied Essential Oils and Lipid Extracts

*E. coli* and *L. monocytogenes* are known as the most common causes of bacterial spoilage of meat and meat products [28,42]. In this context, the minimum inhibitory concentration (MIC) and minimum bactericidal concentration (MBC) were determined for these pathogenic microorganisms. The antibacterial potential of selected essential oil (DEO) and lipid extracts (DSE_1_ and DSE_2_) was determined by the microdilution method, and the results are presented in (Table 2). MIC values of DEO, DSE_1_ and DSE_2_ for both tested pathogenic bacteria ranged from 28.41 to 454.45 µL/mL. According to the recommended classification of plant extracts based on MIC values (weak: MIC above 1500 µg/mL; moderate: MIC between 500 and 1500 µg/mL; strong inhibitors: MIC up to 500 µg/mL), certain values potentially classify them into the group of strong inhibitors against *E. coli* and *L. monocytogenes* [38,39]. Comparing the obtained results, DEO showed the highest antibacterial potential against *E. coli* (MIC = 28.41 µL/mL). In contrast, the lipid extracts (DSE_1_ and DSE_2_) showed higher antibacterial potential (MIC = 113.64 µL/mL, for both) against *L. monocytogenes* compared to DEO (MIC = 454.54 µL/mL). When determining the bactericidal potential against *E. coli*, a lower value was determined for DEO and DSE_2_ (MBC = 113.64 µL/mL, for both) compared to DSE_1_ (MBC = 227.27 µL/mL). Conversely, lipid extract DSE_1_ showed higher bactericidal potential (MBC = 227.27 µL/mL) compared to others against *L. monocytogenes*. The strong antimicrobial potential of the selected EOs and lipid extracts is associated with the content of bioactive compounds, primarily high terpene content, in their case. The most dominant compounds are limonene (44.03–46.64%) and carvone (47.62–50.04%) [28,43]. These results align with research indicating a significant antimicrobial potential of DEO, primarily attributed to the high content of terpenes, with carvone and limonene being among the most dominant in the chemical profile [44].

The mechanism of antimicrobial action of EOs and lipid extracts with a high content of terpenoid compounds is based on disrupting the function of the cell membrane, leading to compromised integrity and leakage of its cellular contents, ultimately resulting in cell death [15,18,28,44]. Despite their strong antimicrobial activity, certain deviations in selective potential towards specific pathogens have been observed, which are influenced using different extraction techniques to isolate target extracts. Apart from differences in aroma and composition, deviations in the achieved selective potential (DEO, DSE_1_, and DSE_2_) are conditioned by variations in volatility and solubility. Essential oils are highly volatile at room temperature, unlike lipid extracts. Similarly, EOs are soluble in non-polar solvents (e.g., alcohol and oils), while lipid extracts are easily soluble in non-polar solvents due to their lipid content. These differences are further influenced by the heterogeneity of the substrates in which they are applied, such as beef burgers [15].

### 3.3. Physicochemical Characteristics of Fresh Beef Burgers

#### 3.3.1. pH and Color Parameters

The pH value of fresh beef burgers ranged from 5.51 to 5.65 (Table 3). The values were generally stable across all treatments, except for treatment DSE_2_75, where a significant (*p* < 0.05) increase was observed on the third day of storage. This phenomenon is associated with the production of alkaline compounds such as peptides, amino acids, and amines [45].

The results of experimental measurements of the color of beef burgers are presented in Table 3. At the beginning of the storage period (day 0), a significant difference (*p* < 0.05) was found between treatments in terms of L* value measurements. In this regard, the highest L* value was measured in the control (C) sample, while the lowest value was recorded in the treatment DEO75, with no significant (*p* > 0.05) difference found among the other treatments. Over time, there was a continuous decrease in L* values in all treatments except for the DEO75 treatment, where an increase was observed, which also represents the highest measured value at the end of the storage period (the third day). This phenomenon contradicts the findings of the study by Trujillo-Santiago et al. [46] Based on the results, it can be concluded that the application of DEO, DSE_1_ and DSE_2_ did not have a significant (*p* > 0.05) effect on the lightness of fresh beef burgers.

In all samples, including the control, a significant (*p* < 0.05) decrease in the (a*, b*) color parameter values of fresh beef burgers was recorded over the storage period. Specifically, in the DSE_1_75; DSE_2_75; DSE_2_150 treatments, a progressive decrease in the a* values was recorded over the storage time, and on the third day, the values were significantly (*p* < 0.05) lower compared to the other treatments. This phenomenon could be the result of lipid oxidation [47]. Additionally, the reduction in a* values is associated with the oxidation of myoglobin and the formation of brown pigment marked as metmyoglobin [48]. During the experimental determination of the red color component on the third day, although there was no significant (*p* > 0.05) difference compared to the remaining samples, it is important to emphasize that the highest a* value was measured in treatment DSE_1_150. In comparison to the previous findings, there was a gradual decrease in the value of the color parameter b* throughout the storage time, but without a significant difference (*p* > 0.05) at the end of the experimental period in all samples.

#### 3.3.2. Oxidative Stability of Beef Burger

**Figure 1:**
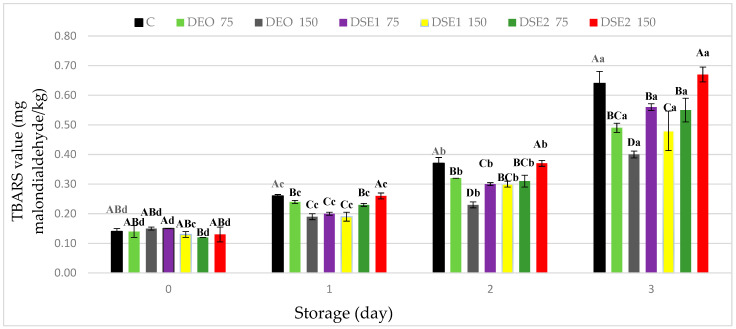
TBARS values (mg malondialdehyde/kg) in fresh beef burger during cold storage. Different upper cases in superscripts (^A–D^) indicate difference (*p* < 0.05) between treatments. Different lower cases superscripts (^a–d^) indicate difference (*p* < 0.05) between days of storage.

The TBARS test, despite its simplicity, represents one of the most relevant methods for examining the degree of lipid oxidation in meat and meat products (Figure 1). At the beginning of the experimental period (day 0), the measured TBARS values did not significantly differ (*p* > 0.05) and ranged from 0.12 mg MDA/kg (DSE_2_75) to 0.15 mg MDA/kg (DEO150 and DSE_1_75) (Figure 1). As expected, over the storage period, TBARS values significantly (*p* < 0.05) increased in all samples, including the control (C). By the end of the experimental period (the third day), the TBARS values significantly (*p* < 0.05) differed among the treatments, and their order from highest to lowest likelihood was DSE_2_150 ≥ C > DSE_1_75 ≥ DSE_2_75 ≥ DEO75 ≥ DSE_1_150 > DEO150. Based on the analysis of the obtained results, it was determined that the TBARS values of the DEO75, DEO150, and DSE_1_150, treatments were lower than the established threshold (≤0.5 mg MDA/kg) which is detectable by consumers [2,49].

The achieved antioxidant effect of lipid extracts aligns with data from the literature. Lipophilic extracts isolated from various plant materials (*Salvia officinalis* L., *Piper auritum Kunth*, *Brosimum gaudichaudii*, *Sizigium aromaticum*, *Ocimum basilicum*, *Cassia corymbose*, *Thymus serpyllum*) have demonstrated exceptional potential in preventing the oxidative stability of minced meat products [3,46,48,50]. On the other hand, in the case of other samples, including the control (C), despite the detected initial rancidity, lower values than the established threshold (1.0 mg MDA/kg), which is considered an indicator of unpleasant taste perception (off-flavor) by sensory analysis [48], were measured.

Based on the results of the TBARS test, it was observed that with the increase in the concentration of the applied lipid extract, from 0.075 µL/g (DEO75, DSE_1_75) to 0.15 µL/g (DEO150, DSE_1_150), the oxidative stability of the present lipids also increased. It is important to emphasize that with the DSE_2_ type of lipid extract, pro-oxidative action was recorded even at a lower applied concentration (DSE_2_75), intensifying with the increase in the applied concentration (DSE_2_150), which is in line with the findings from the study of de Oliveira et al. [48]. Specifically, in the study of the application of the plant extract *Pyrostegia venusta* (PV) and its influence on the oxidative stability of beef burgers, the measured TBARS values of this treatment were higher compared to the control (C) (0.68 and 0.45 mg MDA/kg, respectively). The pro-oxidative action of lipid extracts is associated with the presence of a higher content of co-extracted lipids, primarily polyunsaturated fatty acids (PUFAs) [15,22].

When it comes to the prevention of lipid oxidation, based on the results of this study, it can be concluded that DEO and DSE_1_ have achieved a stronger potential compared to DSE_2_. It is important to emphasize that the selected lipophilic extract DEO, applied at a concentration of 0.075 µL/g, achieved a slightly weaker antioxidant effect in beef burgers compared to DSE_1_ applied at twice the concentration (0.15 µL/g), as evident from the TBARS values (Figure 1) (0.49 and 0.48 mg MDA/kg, respectively). The most effective outcome result in preventing lipid oxidation was achieved by the DEO150 treatment, which had the lowest (*p* < 0.05) measured TBARS value (on the third day) (0.4 MDA/kg). By examining the chemical profile of the selected EO and lipid extracts (DEO, DSE_1_, and DSE_2_), it should be noticed that the differences in the amounts of isolated dominant bioactive compounds (limonene 44.03–46.64% and carvone 47.62–50.04%) were negligible. Therefore, differences in the achieved antioxidant potential can be explained by differences in the solubility of the selected types of extracts, as well as their viscosity, volatility, and oxidizability [15,22]. These differences are influenced by the use of different extraction techniques, but they are further influenced by the heterogeneity of the substrates into which the lipid extracts are incorporated, such as beef burgers.

The reduction of lipid oxidation in the DEO75, DEO150, and DSE_1_150 treatments is associated with the chemical profile of the lipid extracts, and more precisely, the content of dominant bioactive compounds, primarily terpenes (carvone and limonene). The mechanism of the antioxidant action of limonene and carvone involves several processes. Their ability to donate H^+^ ions helps stabilize and neutralize free radicals, which contributes to the prevention of cell membrane damage and the occurrence of cell oxidative stress. They also have the ability to chelate metal ions, inhibit the formation of free radicals, and exclude them from oxidative reactions [51,52].

Myofibrillar proteins, primarily myosin, oxidized lipids, and metal ions, are the main initiators of protein oxidation. During the oxidation of myosin, disulfide and non-disulfide cross-links are formed as products [7,53,54]. Highly reactive compounds (e.g., ROS) are formed during the lipid oxidation process, which, in reactions with cysteine thiol groups, produce various products (e.g., sulfenic, sulfonic acid). The reduction in the total thiol group content serves as an indicator of oxidative protein damage in meat and meat products [7,55].

**Figure 2:**
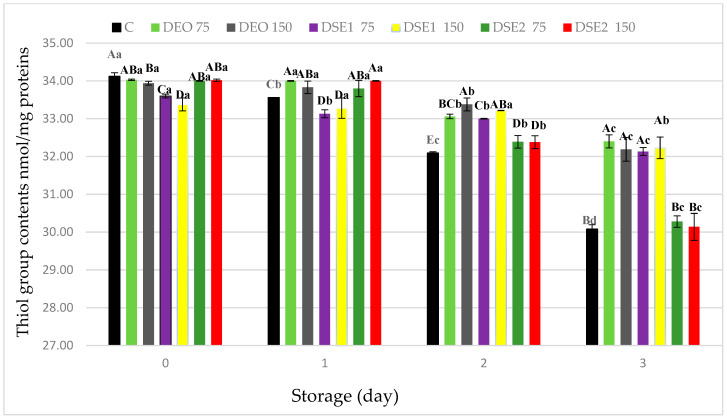
Protein thiol content of in fresh beef burger during cold storage. Different upper cases in superscripts (^A–E^) indicate difference (*p* < 0.05) between treatments. Different lower cases superscripts (^a–d^) indicate difference (*p* < 0.05) between days of storage.

At the commencement of the experimental period (day 0), the thiol group content significantly (*p* < 0.05) differed among treatments (Figure 2).

As anticipated, the thiol group content significantly (*p* < 0.05) decreased over time in all samples, including the control (C). The presented results are consistent with the literature data from the same group of minced meat products [2,31,56]. At the end of the experimental period (the third day), the thiol group content significantly (*p* < 0.05) differed among treatments, and their order from highest to lowest values appears as follows: DEO75 ≥ DSE_1_150 ≥ DEO150 ≥ DSE_1_75 > DSE_2_75 ≥ DSE_2_150 ≥ C. Based on the presented results in this study, which are correlated with TBARS values, it was observed that treatments DEO75, DEO150, DSE_1_75, and DSE_1_150 had significantly (*p* < 0.05) higher thiol group contents compared to the other samples. These results suggest that the DEO75 treatment showed the highest, while the DSE_2_150 treatment showed the lowest potential in preventing oxidative stability of proteins in beef burgers. Regarding lipid oxidation, it was observed that natural ingredients DEO and DSE_1_ have strong antioxidant potential against protein oxidation, attributable to their chemical profile and dominant bioactive compounds, specifically compounds of terpene structure (limonene and carvone) [7,51,57].

### 3.4. Microbiological Quality of Beef Burger

At the beginning of the experimental period (day 0), the TAMB counts ranged from 4.65 (DSE_2_75) to 5.58 log CFU/g (DSE_2_150) (Table 4). As expected, the TAMB counts significantly (*p* < 0.05) increased during the storage period for all samples. At the end of the experimental period (the third day), although the TAMB count values were below 7 log CFU/g, it is important to emphasize that the control sample (C) recorded a significantly (*p* < 0.05) higher value compared to the other treatments. In this regard, the differences in the achieved antimicrobial potential towards TAMB, presented from the highest to the lowest, were DSE_1_150 > DEO75 > DEO150 > DSE_2_75 > DSE_2_150 > DSE_1_75. It is important to note that all natural ingredients (DEO, DSE_1_, DSE_2_) exhibited significant (*p* < 0.05) antimicrobial potential compared to TAMB count (Table 4).

Regarding the microbiological quality of beef burgers (LAB and EB count), the values increased (*p* < 0.05) during the storage period for all samples, including the control (C) (Table 4). DEO and DSE_1_ contributed to the reduction (*p* < 0.05) in EB and LAB, while the addition of DSE_2_ did not have an effect (*p* > 0.05) on the reduction in these bacteria. DEO showed stronger antimicrobial potential towards LAB, while DSE_1_ showed stronger antimicrobial potential towards EB.

In general, the antimicrobial potential of DEO and DSE_1_ is a result of the presence of bioactive compounds, predominantly compounds of a terpene structure. DEO and dill lipid extracts (DSE_1_ and DSE_2_), due to their lipophilic nature, have the ability to bind and penetrate the cell wall and then the cell membrane, taking over and disrupting its multiple functions (e.g., regulation of transport, maintenance of homeostasis, structural support, etc.), which can ultimately lead to cell death [44,58,59]. In the chemical profile of DEO and DSE_1_, carvone and limonene were isolated as dominant terpene compounds (Table 1), which are known for their exceptional antimicrobial potential [51,52,59].

As for DSE_2_, its weak antimicrobial activity is explained by the presence of coextracted lipids, which did not show sufficient solubility in this medium, and the present bioactive components did not achieve the expected antimicrobial effect, especially towards LAB and EB.

The antimicrobial potential of EOs and lipid extracts isolated from selected aromatic plants (fennel; sage; bay leaf; clove, basil, cassia, and thyme; winter savory) has been the subject of numerous studies (respectively), in which their exceptional potential in the same type of minced meat products has also been proven [2,3,10,30,50].

### 3.5. Biogenic Amines Content in Beef Burger

**Figure 3:**
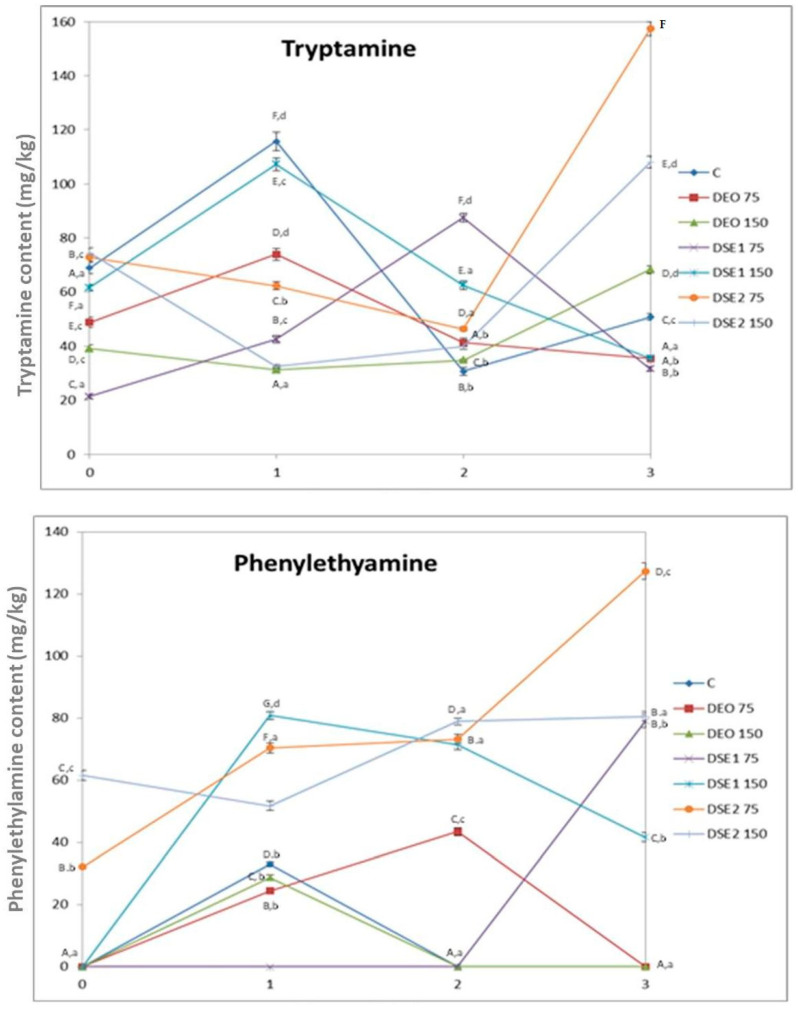

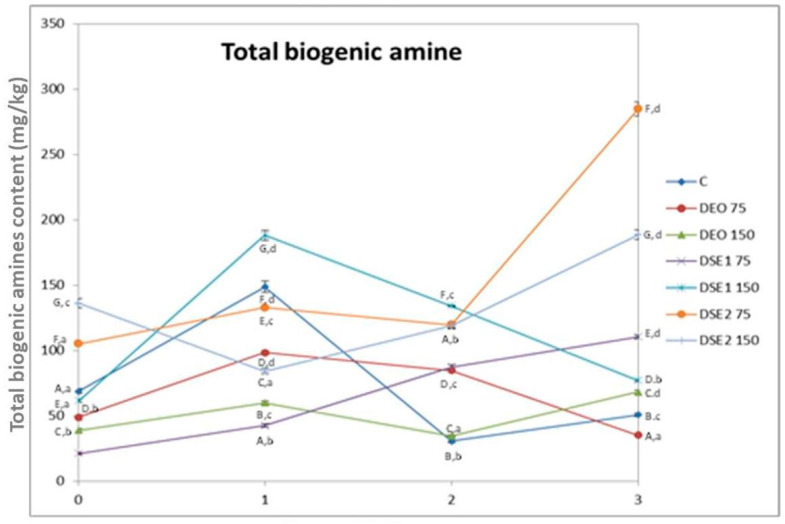
Biogenic amines contents in beef burgers. Different upper cases in superscripts (^A–G^) indicate difference (*p* < 0.05) between treatments. Different lower cases in superscripts (^a–d^) indicate difference (*p* < 0.05) between days of storage.

The content of six biogenic amines in beef burgers was determined using HPLC method for analysis (Figure 3). Putrescine, cadaverine, histamine, and tyramine were not detected in any of the analyzed samples during the storage period. The absence of histamine is particularly important from a toxicological and food safety point of view, given its well-known toxicity. Additionally, it is noteworthy that the Biogenic Amine Index (BAI), calculated from the sum of putrescine, cadaverine, histamine and tyramine, was below the limit of detection. This observation implies that meat is fresh and of good quality, and that good hygiene and manufacturing practices were applied during the whole production and storage period [12].

Tryptamine was determined in all analyzed samples of beef burgers with concentrations ranging from 21.4 mg/kg (DSE_1_75, day 0) to 157 mg/kg (DSE_2_75, day 3), while phenylethylamine was determined in 16 out of 28 samples displaying concentrations ranging from 24.4 mg/kg (DEO75, first day) to 127 mg/kg (DSE_2_75, third day) (Figure 3). The concentrations of these two biogenic amines vary differently within different treatments during the storage period, with similar trends in DEO75 and DSE_1_150. Initially, there was an increase on the first day, followed by a subsequent decrease until the end of the storage period.

The total content of biogenic amines ranged from 21.4 mg/kg to 285 mg/kg, with the maximum level reached on the third day of storage in DSE_2_75 treatment and minimum level reached on day 0 of storage in DSE_1_75 treatment. Similar to tryptamine and phenylethylamine, the total content of biogenic amines exhibited varying trends of decreasing and increasing during the storage period between treatments, with clearly evident potential to reduce the total content of biogenic amines after first day of storage and treatment with DEO75 and DSE_1_150.

## 4. Conclusions

In vitro tests of antimicrobial activity (MIC and MBC) against the most common meat pathogens (*E. coli* and *L. monocytogenes*) have indicated the strong antimicrobial potential of the selected natural ingredients. Examining the chemical profile of dill essential oil and lipid extracts (DEO, DSE_1_, and DSE_2_), it is evident that they possess nearly uniform presence of dominant bioactive compounds, with limonene ranging from 44.03 to 46.64% and carvone from 47.62 to 50.04%. Despite this, a notable difference in achieved antioxidant and antimicrobial potential has been observed and explained. Dill lipid extracts (DEO and DSE_1_) demonstrated strong antioxidant potential towards lipids and proteins. The result of the study, in which the lipid extract DSE_2_ exhibited pro-oxidative effects, will be useful in future research aimed at optimizing the parameters of extraction method, using SFE with CO_2_. DEO and DSE_1_ have shown significant antimicrobial potential against EB and LAB. In this regard, DEO exhibited stronger antimicrobial potential against LAB, while DSE_1_ was more potent against EB. All plant extracts (DEO, DSE_1_, and DSE_2_) displayed significant antimicrobial potential against TAMB. Based on the results of the study, it can be concluded that DEO and DSE_1_ could potentially be used in the production of beef burgers as natural antioxidants and antimicrobial agents.

## Figures and Tables

**Table 1:** Chemical composition of dill essential oil and lipid extracts.

Compound	RT (min)	DEO		DSE_1_		DSE_2_	
		RP (%)	Std	RP (%)	Std	RP (%)	Std
α-Pinene	3.883	tr		tr		tr	
Myrcene	5.075	0.18	0.02	0.19	0.01	0.18	0.02
α-Phellandrene	5.408	1.39	0.01	1.50	0.03	1.54	0.08
*p*-Cymene	5.954	0.30	0.01	0.25	0.02	0.28	0.01
Limonene	6.044	46.26	0.54	44.03	0.04	46.64	0.96
*ρ*-Cymenene (dehydro *p*-Cymene)	7.934	tr		tr		tr	
*cis*-Limonene oxide	9.422	0.12	0.02	tr		tr	
*trans*-Limonene oxide	9.597	tr		tr		tr	
Dill ether	11.376	tr		tr		tr	
α-Terpineol (*p*-Menth-1en-8-ol)	11.615	tr		tr		tr	
*cis*-Dihydro carvone (*cis*-*p*-Menth-8-en-2-one)	11.789	1.13	0.05	1.14	0.02	1.35	0.09
*trans*-Dihydro carvone (*trans*-*p*-Menth-8-en-2-one)	12.049	2.69	0.04	2.84	0.04	2.38	0.08
iso-Dihydro carveol	12.584	tr		tr		nd	
*trans*-carveol	12.758	tr		tr		nd	
neoiso-Dihydro carveol	13.092	tr		tr		nd	
*cis*-carveol	13.214	tr		tr		nd	
Carvone	13.611	47.91	0.49	50.04	0.06	47.62	0.89
Monoterpene hydrocarbons		48.13		45.97		48.64	
Sesquiterpene hydrocarbons		0.0		0.0		0.0	
Oxygenated monoterpenes		51.85		54.02		51.35	
Oxygenated sesquiterpenes		0.0		0.0		0.0	
Other		0.0		0.0		0.0	
Total		99.98		99.99		99.99	

RT—retention time (min); tr—trace; RP—relative percentage (%); nd—not detected.

**Table 2:** Minimal inhibitory concentration (MIC) and minimal bactericidal concentration (MBC) of the dill essential oil and lipid extracts.

	Test Microorganism			
	*Escherichia coli*		*Listeria monocytogenes*	
	MIC (µL/mL)	MBC (µL/mL)	MIC (µL/mL)	MBC (µL/mL)
**DEO**	28.41	113.64	454.54	>454.54
**DSE_1_**	113.64	227.27	113.64	227.27
**DSE_2_**	56.82	113.64	113.64	454.54

**Table 3:** pH and experimental color parameters (L*, a*, b*) changes in fresh beef burger during cold storage.

	**pH**						
Storage day	**Treatments**						
	**C**	**DEO75**	**DEO150**	**DSE_1_75**	**DSE_1_150**	**DSE_2_75**	**DSE_2_150**
0	5.55 ±0.01 ^bABC^	5.56 ±0.01 ^aA^	5.54 ±0.01 ^aBC^	5.55 ±0.01 ^cAB^	5.55 ±0.01 ^bcABC^	5.54 ±0.01 ^bC^	5.54 ±0.01 ^bBC^
1	5.60 ±0.02 ^aC^	5.60 ±0.02 ^bC^	5.57 ±0.01 ^bBC^	5.59 ±0.01 ^aC^	5.53 ±0.01 ^cA^	5.56 ±0.02 ^bB^	5.58 ±0.01 ^aBC^
2	5.58 ±0.03 ^abC^	5.62 ±0.01 ^bA^	5.58 ±0.02 ^bC^	5.54 ±0.01 ^cB^	5.58 ±0.03 ^abC^	5.56 ±0.02 ^bBC^	5.57 ±0.01 ^aBC^
3	5.58 ±0.01 ^abC^	5.59 ±0.02 ^bC^	5.59 ±0.02 ^bC^	5.51 ±0.01 ^bB^	5.58 ±0.01 ^aC^	5.65 ±0.02 ^aA^	5.53 ±0.02 ^bB^
	L*						
Storage day	**Treatments**						
	**C**	**DEO75**	**DEO150**	**DSE_1_75**	**DSE_1_150**	**DSE_2_75**	**DSE_2_150**
0	47.32 ± 3.08 ^aA^	42.60 ± 2.32 ^abB^	46.45 ± 5.77 ^aAB^	45.98 ± 2.46 ^aAB^	46.10 ± 3.34 ^bAB^	44.62 ± 5.87 ^aAB^	45.82 ± 3.43 ^aAB^
1	42.11 ± 3.75 ^bA^	42.11 ± 2.48 ^abA^	42.98 ± 3.83 ^abA^	43.70 ± 3.32 ^aA^	42.71 ± 4.85 ^abA^	41.64 ± 2.58 ^aA^	41.96 ± 4.78 ^aA^
2	40.98 ± 3.44 ^bA^	40.61 ± 3.67 ^bA^	42.06 ± 1.86 ^bA^	42.46 ± 4.66 ^aA^	40.02 ± 2.26 ^aA^	42.64 ± 3.26 ^aA^	42.95 ± 3.54 ^aA^
3	44.02 ± 4.63 ^abA^	44.90 ± 4.19 ^aA^	43.94 ± 2.28 ^abA^	43.75 ± 4.96 ^aA^	43.93 ± 4.16 ^bA^	43.44 ± 3.74 ^aA^	44.21 ± 5.59 ^aA^
	a*						
Storage day	**Treatments**						
	**C**	**DEO75**	**DEO150**	**DSE_1_75**	**DSE_1_150**	**DSE_2_75**	**DSE_2_150**
0	22.17 ± 2.86 ^cB^	23.51 ± 3.73 ^aB^	19.42 ± 2.77 ^aA^	23.17 ± 2.50 ^aB^	22.34 ± 2.75 ^aB^	21.55 ± 3.61 ^aAB^	22.31 ± 1.37 ^aB^
1	21.16 ± 2.35 ^cB^	19.53 ± 2.77 ^cBC^	19.41 ± 2.69 ^aBC^	20.08 ± 3.16 ^bBC^	19.78 ± 2.69 ^bBC^	18.00 ± 1.90 ^bC^	15.67 ± 2.00 ^bA^
2	18.25 ± 1.96 ^aB^	16.99 ± 1.45 ^cBC^	14.29 ± 2.60 ^bDE^	15.05 ± 2.76 ^cCDE^	15.34 ± 1.84 ^cCD^	13.06 ± 2.40 ^cE^	10.71 ± 1.66 ^cA^
3	13.84 ± 3.27 ^bB^	13.49 ± 2.91 ^bB^	13.26 ± 2.32 ^bB^	9.63 ± 2.10 ^dA^	14.56 ± 3.20 ^cB^	8.70 ± 1.93 ^dA^	7.62 ± 0.95 ^dA^
	b*						
Storage day	**Treatments**						
	**C**	**DEO75**	**DEO150**	**DSE_1_75**	**DSE_1_150**	**DSE_2_75**	**DSE_2_150**
0	12.62 ± 1.12 ^abC^	13.15 ± 1.45 ^aBC^	11.63 ± 0.74 ^abA^	13.72 ± 1.10 ^aB^	12.97 ± 0.56 ^bBC^	12.63 ± 0.84 ^aC^	13.20 ± 0.72 ^aBC^
1	13.10 ± 1.01 ^aB^	12.77 ± 1.48 ^abB^	12.51 ± 1.15 ^aB^	13.08 ± 1.32 ^abB^	12.48 ± 1.60 ^bB^	12.15 ± 0.95 ^abAB^	11.16 ± 1.31 ^bA^
2	11.99 ± 1.20 ^bcB^	11.49 ± 0.47 ^bcAB^	11.67 ± 1.49 ^abAB^	12.07 ± 1.67 ^bcB^	10.71 ± 0.59 ^aA^	10.90 ± 1.22 ^bAB^	11.00 ± 1.03 ^bAB^
3	11.31 ± 0.83 ^cA^	11.06 ± 2.05 ^cA^	11.12 ± 1.25 ^bA^	11.18 ± 1.41 ^cA^	12.36 ± 2.16 ^bA^	11.47 ± 2.16 ^abA^	11.24 ± 1.34 ^bA^

Values with different letters ^(A–E)^ in the same row are significantly different (*p* < 0.05); values with different letters ^(a–d)^ in the same column are significantly different (*p* < 0.05).

**Table 4:** Microbiological profile of fresh beef burger during cold storage.

**TAMB (log cfu/g)**							
**Storage day**	**Treatments**						
	**C**	**DEO75**	**DEO150**	**DSE_1_75**	**DSE_1_150**	**DSE_2_75**	**DSE_2_150**
**0**	5.51 ± 0.45 ^Aa^	5.51 ± 0.39 ^Aa^	4.78 ± 0.08 ^Ba^	4.74 ± 0.04 ^Bb^	4.72 ± 0.01 ^Ba^	4.65 ± 0.05 ^Bb^	5.58 ± 0.01 ^Aa^
**1**	5.39 ± 0.09 ^Aa^	4.34 ± 0.09 ^Cc^	4.30 ± 0.07 ^CDc^	4.32 ± 0.02 ^CDd^	4.34 ± 0.09 ^Cb^	4.22 ± 0.01 ^Db^	4.64 ± 0.04 ^Bc^
**2**	5.11 ± 0.03 ^Aa^	4.90 ± 0.01 ^Bb^	4.35 ± 0.05 ^Dc^	4.51 ± 0.03 ^Cc^	4.34 ± 0.04 ^Db^	4.09 ± 0.09 ^Ec^	4.83 ± 0.01 ^Bb^
**3**	5.45 ± 0.03 ^Aa^	4.23 ± 0.03 ^Ec^	4.62 ± 0.02 ^Db^	4.94 ± 0.03 ^Ba^	4.11 ± 0.07 ^Fc^	4.73 ± 0.03 ^Ca^	4.79 ± 0.01 ^Cb^
**LAB (log cfu/g)**							
**Storage day**				**Treatments**			
	**C**	**DEO75**	**DEO150**	**DSE_1_75**	**DSE_1_150**	**DSE_2_75**	**DSE_2_150**
**0**	3.98 ± 0.01 ^Aa^	3.62 ± 0.02 ^Ba^	2.74 ± 0.04 ^Eb^	2.81 ± 0.04 ^Db^	2.72 ± 0.02 ^Eab^	2.93 ± 0.07 ^Cc^	2.49 ± 0.01 ^Fc^
**1**	3.87 ± 0.03 ^Ab^	2.35 ± 0.01 ^CDc^	2.09 ± 0.09 ^Dd^	3.13 ± 0.02 ^Ba^	2.51 ± 0.03 ^Cc^	3.31 ± 0.01 ^Bb^	3.00 ± 0.48 ^Bb^
**2**	3.38 ± 0.02 ^Ac^	2.32 ± 0.02 ^Cc^	2.30 ± 0.00 ^Cc^	2.58 ± 0.28 ^BCb^	2.63 ± 0.15 ^BCbc^	2.38 ± 0.38 ^Cd^	2.75 ± 0.03 ^Bbc^
**3**	3.83 ± 0.05 ^Bb^	2.64 ± 0.04 ^Fb^	2.96 ± 0.00 ^Da^	3.33 ± 0.03 ^Ca^	2.81 ± 0.03 ^Ea^	3.80 ± 0.02 ^Ba^	3.96 ± 0.00 ^Aa^
**EB (log cfu/g)**							
**Storage day**				**Treatments**			
	**C**	**DEO75**	**DEO150**	**DSE_1_75**	**DSE_1_150**	**DSE_2_75**	**DSE_2_150**
**0**	2.24 ± 0.06 ^Ab^	1.93 ± 0.03 ^Bb^	1.30 ± 0.00 ^Cc^	1.15 ± 0.15 ^Cc^	1.15 ± 0.15 ^Cb^	1.15 ± 0.15 ^Cc^	1.15 ± 0.15 ^Cc^
**1**	2.50 ± 0.02 ^Aa^	1.81 ± 0.03 ^Db^	2.06 ± 0.06 ^Ba^	1.98 ± 0.02 ^Cb^	1.81 ± 0.03 ^Da^	1.87 ± 0.03 ^Db^	2.07 ± 0.07 ^Bb^
**2**	1.93 ± 0.03 ^Ac^	1.15 ± 0.15 ^Cc^	1.74 ± 0.04 ^Bb^	1.90 ± 0.05 ^Ab^	1.00 ± 0.00 ^Db^	1.74 ± 0.04 ^Bb^	1.90 ± 0.05 ^Ab^
**3**	2.54 ± 0.06 ^Aa^	2.31 ± 0.01 ^Ba^	2.06 ± 0.02 ^Ca^	2.25 ± 0.02 ^Ba^	1.15 ± 0.15 ^Db^	2.53 ± 0.05 ^Aa^	2.59 ± 0.11 ^Aa^

Values with different letters ^(A–F)^ in the same row are significantly different (*p* < 0.05); values with different letters ^(a–d)^ in the same column are significantly different (*p* < 0.05).

## Data Availability

The original contributions presented in the study are included in the article, further inquiries can be directed to the corresponding author.
